# Absence (Congenital) of the Anterior Lobes of the Brain

**Published:** 1831-01-01

**Authors:** 


### XXVII.

### Absence (Congenital) of the Anterior Lobes of the Brain.

In the annual report of the new Anatomical Society of Paris, a preparation was shewn by M. Lacroix, exemplifying the above mal-organization. The secretary of the society makes use of the following words: "if the opinion which assigns to the anterior lobes of the brain the privilege of presiding over the higher intellectual operations, needed any new confirmation, it would find a powerful argument in its favour in the case reported by M. Lacroix. In that case there was a complete congenital absence of the anterior lobes of the cerebrum, which were replaced by a collection of transparent serum communicating freely with the ventricles. This physical condition was accompanied, not by a perversion, but by an almost entire nullity of the intellectual and moral functions. Here was an experiment made by nature, more valuable for physiology than any vivisections of the anatomist." The secretary remarks that this case tells both for and against the phrenologists—for them, as shewing the seat of intelligence to be in the anterior part of the brain — against them, as shewing that their skill could not have detected the cause of the idiocy, since the forehead was well-formed, though full of water, and all the prominences well-marked.

Almost at the same time that the above preparation was shewn, another came under view where the left hemisphere of the brain was found atrophied to one half its original volume, without any loss of intellectual faculties, the other lobe being entire. The atrophy was occasioned by an accumulation of fluid in the lateral ventricle of that side, and the opposite half of the body was completely paralytic.

---

